# P06-04 Agreement between single item physical activity measures in population surveillance in New Zealand

**DOI:** 10.1093/eurpub/ckac095.089

**Published:** 2022-08-29

**Authors:** Bauman Adrian, Anja MIzdrak, Ryan Gage, Hamish McEwan, Justin Richards

**Affiliations:** 1 School of Public Health, Sydney University, Sydney NSW, Australia; 2 Public Health, University of Otago, Wellington, New Zealand; 3 Manager, Sport New Zealand, Wellington, New Zealand; 4 Associate Professor, Victoria University, and Senior Adviser, Sport New Zealand, Wellington, New Zealand

**Keywords:** physical activity, single item, sport, surveillance, epidemiology

## Abstract

**Background:**

Sport New Zealand conducts continuous physical activity and sport “Active NZ” surveys, conducted since 2017; these survey around 20,000 representatively sampled adults annually (response rate 29-32%, data weighted to NZ Census population). There has been international interest in “single item” physical activity questions to estimate and monitor physical activity levels. Validated questions for adults have asked about the number of days in the past week people were active for > =30 mins for leisure or transport (SI-days). The Active NZ surveys also ask IPAQ-long form questions and a new single item question on the number of hours people were active in sport/recreation in the past 7 days (SI-hours). The public health problem is that the validated SI-days question cannot directly estimate the WHO recommended threshold of 150+ mins of PA/week. This study describes the prevalence of these questions, and examines the relationships between SI-days, SI-hours and IPAQ-long form [using 600 total PA met-mins as the threshold approximating 150 mins of moderate PA, and also using the leisure time domain only in IPAQ).

**Methods:**

Analyses were descriptive, and inter-method comparisons using Spearman's correlations, and the best fit with the PA thresholds were estimated using Area under the ROC curve (AUC) and Youden's Index.

**Results:**

IPAQ and SI-days were only collected mid 2019 to early 2020 (n = 15044). SI-days showed a mean of 3.2 days (*SD*=2.2), SI-hours 5.3 (*SD*=6.2), and SI-hours 150 mins+ was reported by 60.6%. 88.2% reached the IPAQ-total met threshold, and 41.4% met the IPAQ leisure only threshold. Correlations between SI measures and IPAQ were 0.22 for IPAQ-total, and ∼ 0.45 for IPAQ-leisure. SI-days and SI-hours were correlated rho=0.51. ROC curve analyses showed AUC values with IPAQ measures were between 0.63 to 0.76, but the SI-days showed a good AUC of 0.82 (0.81-0.83) with the SI-hours 150 mins threshold. Youdens index suggested the best fit was at 3+ days/week were most likely to meet the SI-hours threshold.

**Conclusion:**

These data show SI questions reflect health enhancing thresholds well, and that those reporting >3 SI days showed best fit with the 150mins threshold based on SI hours, indicating good surveillance utility.

